# Microbial contamination and composition of oral samples subjected to clinical whole genome sequencing

**DOI:** 10.3389/fgene.2023.1081424

**Published:** 2023-02-07

**Authors:** Abhishek Kumar, Volha Skrahina, Joshua Atta, Veronika Boettcher, Nicola Hanig, Arndt Rolfs, Gabriela Oprea, Najim Ameziane

**Affiliations:** ^1^ Arcensus Diagnostics, Rostock, Germany; ^2^ CeGaT GmbH, Tübingen, Germany; ^3^ Medical Faculty University of Rostock, Rostock, Germany

**Keywords:** WGS, gentic testing, genetic diagnostics, microbial contamination, molecular diagnostics

## Abstract

Biological material from the oral cavity is an excellent source of samples for genetic diagnostics. This is because collection is quick, easy-to-access, and non-invasive. We have set-up clinical whole genome sequence testing for patients with suspected hereditary disease. Beside the excellent quality of human DNA that can be isolated from such samples, we observed the presence of non-human DNA sequences at varying percentages. We investigated the proportion of non-human mapped reads (NHMR) sequenced from buccal swabs and saliva, the type of microbial genomes from which they were derived, and impact on molecular classification. Read sequences that did not map to the human reference genome were aligned to complete reference microbial reference sequences from the National Center for Biotechnology Information’s (NCBI) RefSeq database using Kraken2. Out of 765 analyzed samples over 80% demonstrated more than 5% NHMRs. The majority of NHMRs were from bacterial genomes (average 69%, buccal swabs and 54% saliva), while the proportion of viruses was low, averaging 0.32% (buccal swabs) and 0.07% (saliva). We identified more than 30 different bacterial families of which *Streptococcus mitis* and *Rothia mucilaginosa* were the most common species. Importantly, the level of contamination did not impact the diagnostic yield.

## Introduction

Saliva and buccal samples are increasingly used for medical research studies, including those using modern “omics” platforms. The oral cavity is an excellent source of biological material for genetic and omics studies (Theda et al., 2018). Oral sampling is fast, easy-to-handle, and non-invasive compared to tissues such as blood (Juarez-Cedillo et al., 2010; Langie et al., 2016). Because of the simplicity and safety of the procedure, subjects can collect the samples themselves and samples can be sent by post.

We established a whole genome sequencing (WGS) testing pipeline for genetic diagnosis. While mapping the genomic data, we noticed that several samples have large amounts of reads not mapping to the human reference assembly hg38. Exploration of these reads indicated the presence of microbial DNA. In this study, we systematically examined the microbial composition of samples obtained from buccal swabs and saliva. In addition, we investigated whether the presence of microbial contamination had an impact on the molecular diagnosis of the subjects. A positive diagnosis is considered achieved if 1) a known pathogenic or likely pathogenic variant is identified in a gene that can explain the symptoms in the respective subject or 2) a unique or rare variant (MAF < 0.001%) that is expected, based on multiple *in silico* pathogenicity predictions tools, to have a deleterious effect on transcript expression or protein function in a disease associated gene.

## Results and discussion

The workflow for the identification of non-human mapped reads (NHMR) and their microbial composition from human whole genomic is performed using publicly available tools SAMtools (Li et al., 2009), Kraken2 (Wood et al., 2019) and Pavian (Breitwieser and Salzberg, 2020) and is schematically represented in Supplementary Figure S1.

### Impact of non-human DNA contamination on sequencing statistics

A total of 765 samples were included in this study, of which 653 were from buccal swabs (Pakistan: 537, Albania: 112, Germany: 2, and Middle East: 6) and 112 (Pakistan: 95, Albania: 3, Germany: 8, and Middle East: 2) were from saliva. A median average of 777 million reads were generated per sample that passed Illumina chastity filter (http://www.Illumina.com), resulting in a median whole genome sequencing depth of coverage of 29x. Buccal swabs showed less variability in the total number of reads compared to saliva, while there were outliers with a maximum of 2.8 billion reads and a minimum of 479 million reads (Supplementary Figure S2). These differences can be explained due to pooling procedures prior to loading sample libraries on the sequencing flow cells. As expected, a clear correlation is observed between the total number of reads and the average depth of coverage, where the correlation coefficient is drastically enhanced after considering reads mapping to human reference genome only (Supplementary Figure S2). The percentage of NHMR for buccal swabs and saliva was similar.

**FIGURE 1:**
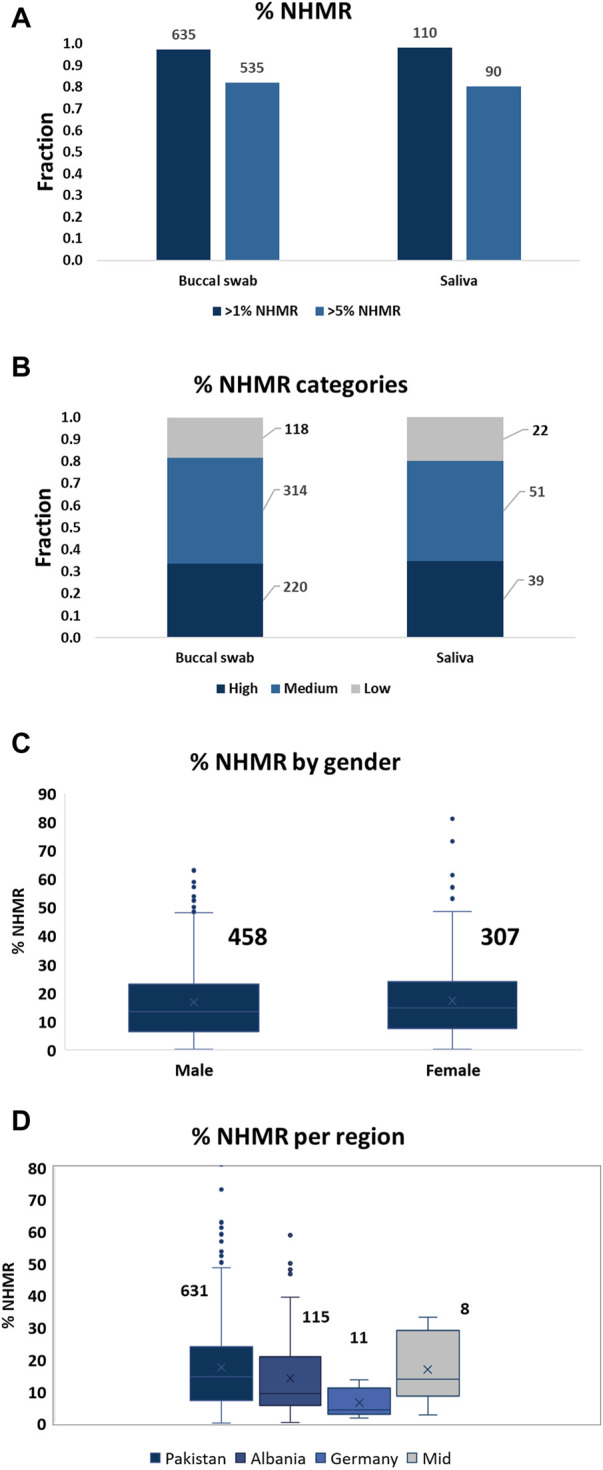
The level of NHMR among the samples, per gender, and per geographical region. **(A)** The distribution of samples with at least 1% or 5% NHMR for buccal swab and saliva samples. The total numbers for the categories are displayed on top of the bars. An unpaired t-test (Mann-Whitney test) demonstrated no significant difference in %NHMR between buccal swab and saliva samples (*p*-value = 0.8). **(B)** The distribution of samples into low (<5% NHMR), medium (5%–20% NHMR) and high (>20% NHMR) categories. The total number of cases per category are displayed on the side. **(C)** The distribution of % NHMR for samples obtained from males and females. The total numbers for the categories are displayed on top of the bars. The difference in %NHMR between males and females is not statistically significant. **(D)** The distribution of %NHMR for the samples from different geographical regions. Pakistan, 631; Albania, 115; Germany, 11; Middle East, 8.

The subject’s gender did not affect the level of NHMR, while a slight difference in NHMRs was observed between samples from different geographical regions. Subjects from Germany demonstrated the lowest percentage of NHMR (Figure 1). However, since only 10 cases from Germany and eight cases from the Middle East were included, the case numbers were too low to establish a solid correlation between region’s sample origin and NHMR.

### Majority of NHMR is of bacterial origin and a minor fraction is of viral origin

Out of a total of 653 buccal swab and 112 saliva samples, 535 (82%) and 90 (80.4%) cases demonstrated more than 5% of NHMR, respectively (Figure 1). The samples were subsequently classified into low (<5% NHMR), medium (5%–20% NHMR) and high (>20% NHMR) NHMR categories (Figure 1; Supplementary Table S1). The median percentage of NHMRs was 13.6% for buccal swabs and 13.3% for saliva samples. The buccal swabs demonstrated extreme outliers with cases of NHMRs of up to 81%, while the highest NHMRs for saliva reached 48%. Absence of extreme outliers for saliva can be explained by the low number of samples compared to the total number of buccal swabs. Indeed, the distribution of the NHMR for buccal swab and saliva samples seem to follow a similar pattern (Supplementary Figure S7).

Most NHMRs were mappable to bacterial genomes with a median average of 69% and 62% of NHMRs for buccal swabs and saliva samples, respectively. Considering at least 5% of NHMR aligning to microbial genomes, most samples demonstrated the presence of more than one bacterial family while in two saliva samples and six buccal swab samples six different bacterial families were detected (Supplementary Table S1). The presence of viral DNA was relatively rare. A median average of 0.05% and 0.03% of NHMRs mapped to viral genomes for buccal swabs and saliva samples, respectively (Supplementary Table S1). Outliers of up to 31.25% and 1.3% NHMRs mapping to viral genomes were observed for buccal swabs and saliva, respectively. Considering at least 0.5% of NHMRs aligning to microbial genomes, which accounts for about 200.000 reads, 23 buccal swab and two saliva samples demonstrated the presence of viral genomes. Expectedly, most of these viral DNA reads originated from bacteriophages (Supplementary Table S1). Exceptionally, some samples have higher viral contents like three buccal swab samples (arc000174 (31,25%), arc000372 (22,5%) and arc000274 (12,73%)) have >10% of NHMRs with viral origins. In all the three cases, the genetic etiology could be identified suggesting that the high viral abundance did not impact the molecular diagnosis.

A median average of almost 32% of the NHMR could not be assigned to any of over 83 thousand addressed bacterial or viral genomes. These reads may fall into any or a combination of the following categories: a) bad quality reads due to sequencing errors that do not allow proper alignment to the interrogated genomes; b) the reads are of human origin, but complex genomic alterations in a sample does not allow proper initial alignment to the reference genome; c) the reference genome(s) of the bacterial and/or viral species from which the read originate are not interrogated by Kraken2; d) the reads originate from microorganisms other than bacteria and viruses, such as fungi and protozoa.

### Streptococcaceae is the most prevalent microbial family with *Streptococcus mitis* as top bacterial species

Using a cut-off of >5% NHMRs and a minimum of 20 samples for which the same bacterial species is observed, Streptococcaceae was the most abundant bacterial family; identified in 611 buccal swab and 78 saliva samples, accounting for 94% and 70% the cases, respectively (Table 1). Other bacterial major families are Neisseriaceae, Pasteurellaceae, Actinomycetaceae, Micrococcaceae, Veillonellaceae and Prevotellaceae. *Streptococcus mitis* is the most prevalent in these samples, when considering >5% NHMRs and a minimum of five samples (Table 1). In previous studies, it was demonstrated that *Streptococcus* species, S*. mutans* (Psoter et al., 2011; Vieira et al., 2011) and *S. parasanguinis* (Mahfuz et al., 2013), are the major source of NHMRs in human oral cavity. Streptococci are the first inhabitants of the oral cavity with acquisition immediately after birth (Abranches et al., 2018). Hence, these bacteria are major components of the oral microbiota. Additionally, Streptococci are causative agents of dental caries including *S. mitis* (Banas et al., 2016). *S. mitis* serve as one of the major components of the oral streptococci classification systems (Whiley and Beighton, 1998; Richards et al., 2014). Oral Streptococci are potent producers of adhesive molecules, which are instrumental in colonizing these microbes within different oral tissues (Abranches et al., 2018). Taken, together, it is evident that oral cavities have a suitable environment for growth of other Streptococci including *S. mitis.*

**TABLE 1:** The prevalence of bacterial families and species among the samples.

	Buccal swab	Saliva
Bacterial families		
Streptococcaceae	611 (94%)	78 (70%)
Neisseriaceae	287 (44%)	53 (47%)
Pasteurellaceae	179 (27%)	31 (28%)
Actinomycetaceae	160 (25%)	19 (17%)
Micrococcaceae	151 (23%)	33 (29%)
Veillonellaceae	143 (22%)	26 (23%)
Prevotellaceae	106 (16%)	43 (38%)
Flavobacteriaceae	70 (11%)	5 (4%)
Corynebacteriaceae	35 (5%)	5 (4%)
Burkholderiaceae	24 (4%)	3 (3%)
Bacterial species		
*Streptococcus mitis*	418 (64%)	36 (32%)
*Streptococcus pneumoniae*	109 (17%)	6 (5%)
*Rothia mucilaginos*	96 (15%)	24 (21%)
*Veillonella parvula*	65 (10%)	6 (5%)
*Prevotella melaninogenica*	39 (6%)	17 (15%)
*Streptococcus oralis*	37 (6%)	2 (2%)

### Microbial composition is variable across samples

**FIGURE 2:**
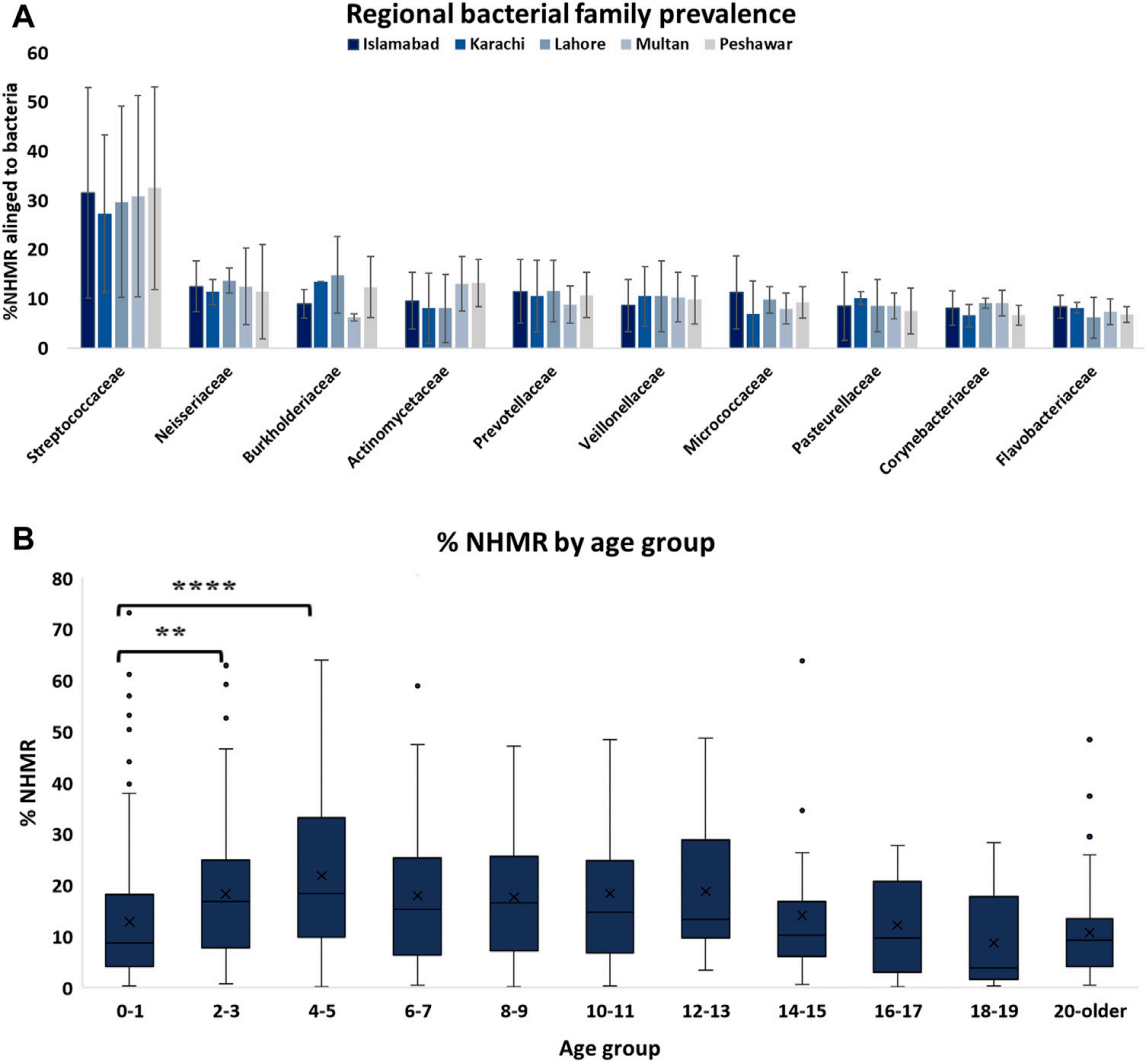
Level of NHMR across age groups and prevalence of bacterial families among samples from different geographical regions. **(A)** The distributions of bacterial family contamination among samples from different geographical regions in Pakistan. **(B)** The level of NHMR within the different age categories. The adjusted *p*-value based on a one-way ANOVA are 0.023 (**) and <0.0001 (****).

The level and composition of microbial species within samples was variable. When considering the top ten most prevalent bacterial families, no significant difference in contamination level from different bacterial families was observed between the samples from the different geographies (Figure 2). An increased level of NHMR was observed for individuals between the age of two and 13 years of age (Figure 2). A significant difference in %NHMR was identified when comparing the first age group (0–1) to age groups 2–3 and 4–5 but not when compared to older age groups. This may be explained by the absence of teeth in children under the first year of age and better oral hygiene for subjects of the age six and older. A diverse bacterial composition within subjects was observed, as is illustrated but not limited to subjects with the highest NHMRs (Supplementary Figure S4). Sample arc000199, with 19.11% NHMRs, has 65.6 million reads (M) aligning to *Ottowia* sp. oral taxon 894. Other major bacterial components prevalent in the same sample (Supplementary Figure S4), are *Bergeyella cardium* (32.1 M), *Rothia mucilaginosa* (22.3 M). The microbial composition for the different samples is shown in Supplementary Figure S3, with *S. mitis* observed in all samples at quantities and the highest abundance in sample arc000427. Buccal swab sample arc000019 has various bacterial families (Supplementary Figure S4) with top three species: *Neisseria subflava* (18.7 M), *Prevotella melaninogenica* (16.1 M), and *Haemophilus parainfluenzae* (9.1 M). Similarly, buccal swab sample arc000204 has major bacterial fragments (Supplementary Figure S4) as *P. melaninogenica* (18.3 M), *P. jejuni* (16.3 M), and *Veillonella parvula* (15.9 M). Saliva-based sample arc000701 has several bacterial components (Supplementary Figure S4), the top three of which are *P. melaninogenica* (6.0 M), *N. subflava* (4.3 M), and *P. intermedia* (4.3 M). Saliva sample arc000743 has major bacterial fragments (Supplementary Figure S4) of which the top three are *N. sicca* (9.99 M), *P. melaninogenica* (9.7 M), and *Fusobacterium nucleatum* (7.3 M).

### High prevalence of beta herpesvirus 5 in buccal swabs

Three buccal swab samples (samples: arc000174 arc000372 and arc000274) demonstrated more than 10% of NHMRs aligning to viral genomes (Supplementary Table S1) compared to an average of 0.32% for all buccal swab samples. The NHMRs in these samples were predominantly aligning to beta-herpesvirus 5 with a total of 4.92, 2.48, and 2.09 M in arc000174, arc000372 and arc000274, respectively (Supplementary Figure S5). When the threshold of NHMRs aligning to viral genomes was lowered to >0.5%, beta-herpesvirus 5 was identified in 28 samples (2 saliva samples and 26 buccal swab samples). Three other prominent viral family genomes that were detected in the samples were Myoviridae (5x), Podoviridae (5x) and Siphoviridae (7x). Notably, these three viral families have bacteria as their natural host, which could explain their high prevalence.

### No impact of microbial contamination on genetic diagnosis

**FIGURE 3:**
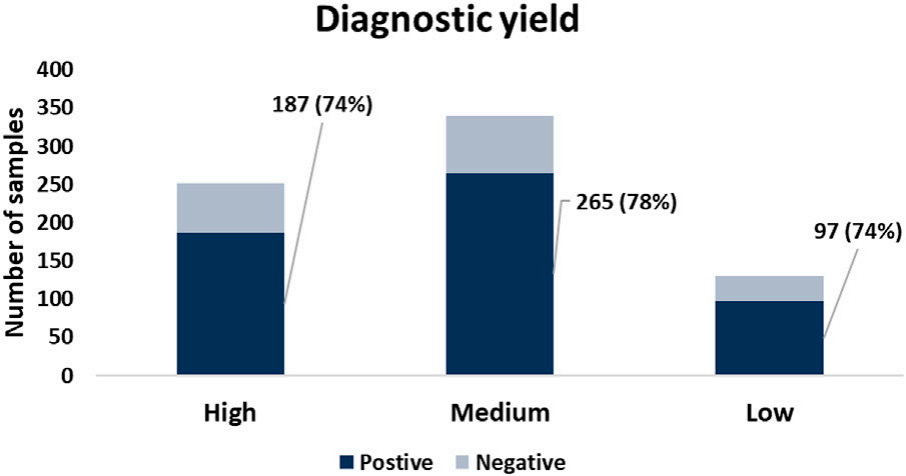
The diagnostic yield among samples with different levels of %NHMR. No significant difference in diagnostic yield observed between groups with different levels of NHMR.

To assess whether the presence of NHMR had impact on the molecular diagnosis of the subjects, we focussed on subjects with either a positive or a negative primary molecular classification. Cases were categorized in low, medium, and high contamination groups based on NHMRs of less than 5% (131), >5–20% (340), and >20% (252), respectively. Remarkably, the diagnostic yield was highly comparable between the low (74%), medium (78%) and high (74%) contamination groups (Figure 3). The chi-square test demonstrated a *p*-value of 0.49, indicating no significant difference between groups. A genetic diagnosis could be established for cases with NHMRs over 70%. Also, for both buccal swab and saliva samples, the level of NHMR did not demonstrate a significant impact on diagnostic yield (unpaired t-test, *p*-values 0.27 and 0.84, respectively. Supplementary Figure S6). The findings for all cases are summarized in Supplementary Table S1. Altogether, although samples collected from the oral cavity for the purpose of clinical molecular diagnostics are often contaminated by bacterial and viral species, the diagnostic yield is not impacted. Due to its safety, simplicity and ease, oral sampling can be considered an excellent DNA collection approach in a clinical diagnostic setting.

In this study, we systematically investigated the origin of non-human mapped reads of samples obtained from the oral cavity that were whole genome sequenced for clinical genetic testing. Over 70% of cases demonstrated the presence of more than 5% microbial contamination. The contamination ranged from a single microbial species to up to six species in a single sample. Nevertheless, the contamination did not negatively impact the genetic diagnosis of the subjects. The reference genomes of the identified highly prevalent microbial species can be added to the human reference genome as decoy in routine clinical diagnostic WGS analysis to improve read alignment and variant calling, especially for samples collected from the oral cavity.

## Methods

### Sample collection

During hospital visits, patients suspected to suffer from a genetic disease were sampled by physicians who, in parallel, carefully evaluated and documented the phenotypes. Consent to participate in research projects was obtained for all subjects. Buccal swabs and saliva samples were collected using DNA ORAcollect (Genotek, Canada) OCR-100 and OG-500 kits, respectively. The participants or guardians of the participating children signed written informed consent forms. The study was approved by the Ethics Committee of Rostock University (Germany), no. A2022-0072, 25.04 2022.

### DNA preparation and WGS

DNA extraction from buccal swabs and saliva was performed using prepIT.L2P kit (DNA Genotek, Canada). DNA libraries for WGS were prepared using TruSeq Nano DNA Library Prep Kit (Illumina) and sequenced with the 150 bp paired-end protocol on an Illumina platform to yield an average coverage depth of 30x for the nuclear genome and at least 1000x for the mitochondrial genome.

### Data analysis

Raw reads were mapped to reference human genome assembly GRCh38. p13 using bwa (version 0.7.17-r1188) software with the mem algorithm (Li and Durbin, 2009). A bam file subset was created containing reads not aligned to human genome (flagged “-f4”) using SAMtools version 1.10^4^ after which these reads were extracted using SAMtools bamtofastq module (Li et al., 2009) to generate paired-end zipped fastq files of non-human mapped reads (NHMR). Next, these fastq files were analyzed with Kraken version 2.0.8-beta (Wood et al., 2019) against the complete reference microbial reference sequences from the National Center for Biotechnology Information’s (NCBI) RefSeq database; minikraken2_v2_8 GB_201904. A complete list of interrogated genomes can be found in Supplementary Table S1. For the visualization of the microbial composition, Kraken2 output files were taken to create Sankey diagrams using Pavian (Breitwieser and Salzberg, 2020). A schematic overview of the procedure is illustrated in Supplementary Figure S1.

## Data Availability

The original contributions presented in the study are publicly available. This data can be found here: https://www.ncbi.nlm.nih.gov/sra/?term=PRJNA917836.

## Supplementary material

Supplementary Figure S1 Schematic overview of the bioinformatic pipeline used to explore microbial presence and composition of samples collected from the oral cavity.

Supplementary Figure S2 Sequencing statistics. (A) The distribution of total reads obtained for buccal swabs and saliva samples. (B) The average depth of coverage distribution for the samples. (C) The correlation between total reads and average depth of coverage before and (D) after considering exclusively reads mapped to human reference genome.

Supplementary Figure S3 Microbial composition of buccal swab samples. The distribution of NHMR aligning to the different bacterial and viral species for samples with the highest percentage of NHMR. The number of reads aligning to the different species are indicated with colored bars as illustrate the frequency among species. The top panel indicates the percentage of reads aligned to the human reference genome GRCh38.p13, starting with 19.11% to 90.41%.

Supplementary Figure S4 Phylogenic tree of microbial composition for samples arc000199 (A), arc000019 (B), arc000204 (C), arc000701 (D), arc000743 (E).

Supplementary Figure S5 Phylogenic tree of microbial composition of buccal swab samples containing high beta herpesvirus 6 levels. D-Domain, K-Kingdom, P- Phylum, C-Class, O-Order, F-Family, G-Genus, and S-Species. The numbers at the nodes indicate the total NHMR aligned at the respective level. (A) Sample arc000174. (B) Sample arc000372. (C) Sample arc000274.

Supplementary Figure S6 The level of NHMR does not affect the diagnostic yield. Box plots showing the distribution of NHMR in percentages (Y-axis) for cases with either a positive or negative molecular diagnostic finding for buccal swab (A) and saliva (B) samples.

Supplementary Figure S7 The NHMR distribution of the collected samples. A density plot demonstrating the distribution of the level NHMR for buccal swab and saliva samples. The percentage of NHMR is plotted on the X-axis.

Supplementary Table S1 Overview of 765 samples collected either by buccal swab (*n* = 653) or saliva (*n* = 112). TotalReads: total number of sequencing reads. averageDP: average whole genome depth of coverage. Reads aligned %: Percentage of reads aligned to human reference genome. %NHMR: percentage of non-human mapped reads. %Bacteria (of NHMR): percentage of NHMR aligned to bacterial genomes. %Viruses (of NHMR): percentage of NHMR aligned to viral genomes. Material: Sample collection method. Country: Country origin of sample. DOB: Date of birth of the individual from whom the sample was collected. YOB: Year of birth. Gender: sex of the sample. Status: Provided clinical status of proband. Diagnostic_Outcome: Whether genetic etiology for proband was detected. NHMR_Cat: The category of NHMR, high/medium/low. Number of Species: number of detected microbial species, which have a minimum of 200 k reads. Top3Species: The top three detected species ordered from most abundant to less abundant.

Supplementary Table S2 Lists of microbial genomes separated in two sheets: A bacterial- and a viral genomes sheet.
