# Supplementary figures and images for: Microbial contamination and composition of oral samples subjected to clinical whole genome sequencing
Source: Front Genet. 2023 Feb 7;14:1081424. doi: 10.3389/fgene.2023.1081424 (PMC9941560; doi:10.3389/fgene.2023.1081424)

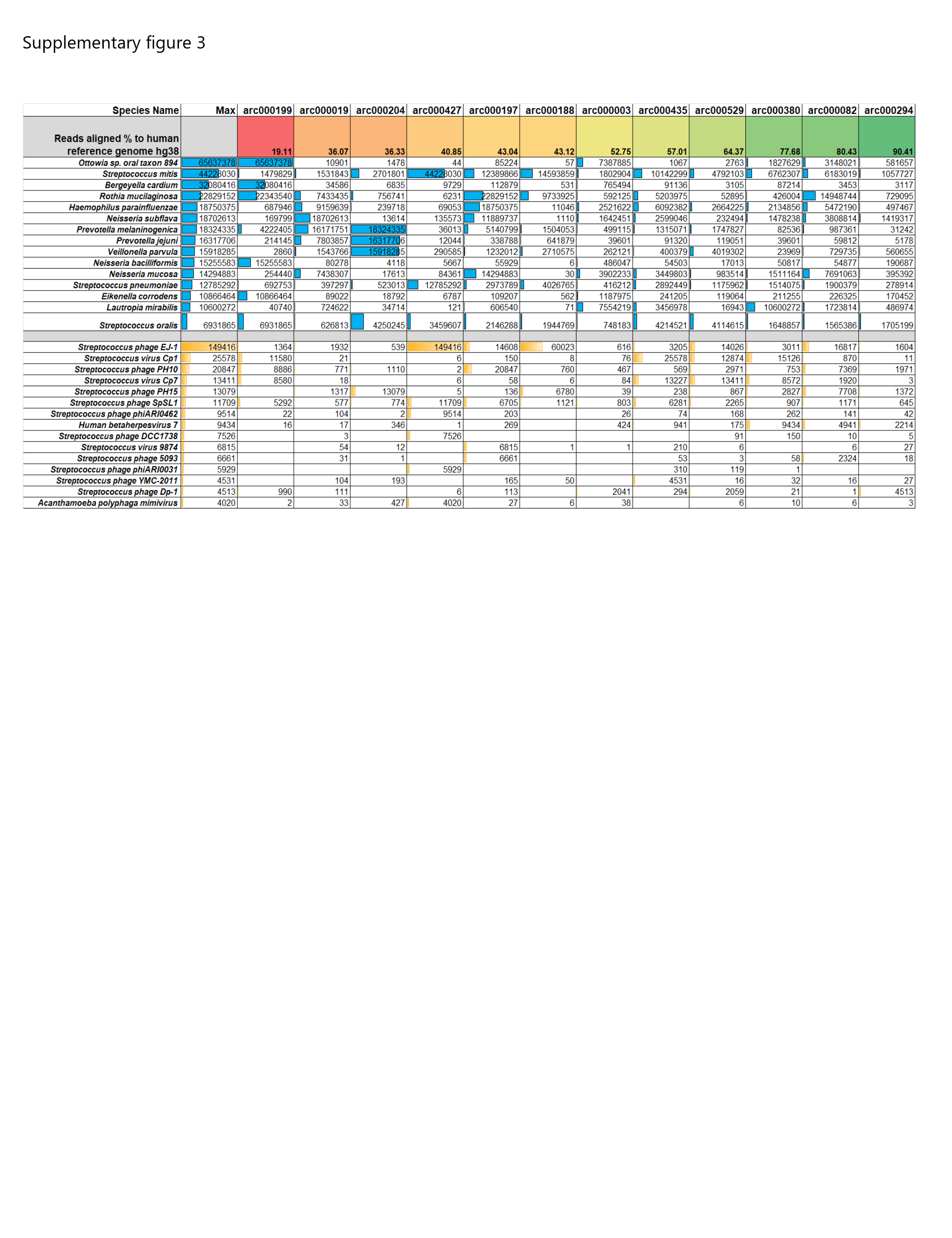

Supplement: Supplementary file 1 [file Image3.TIFF]

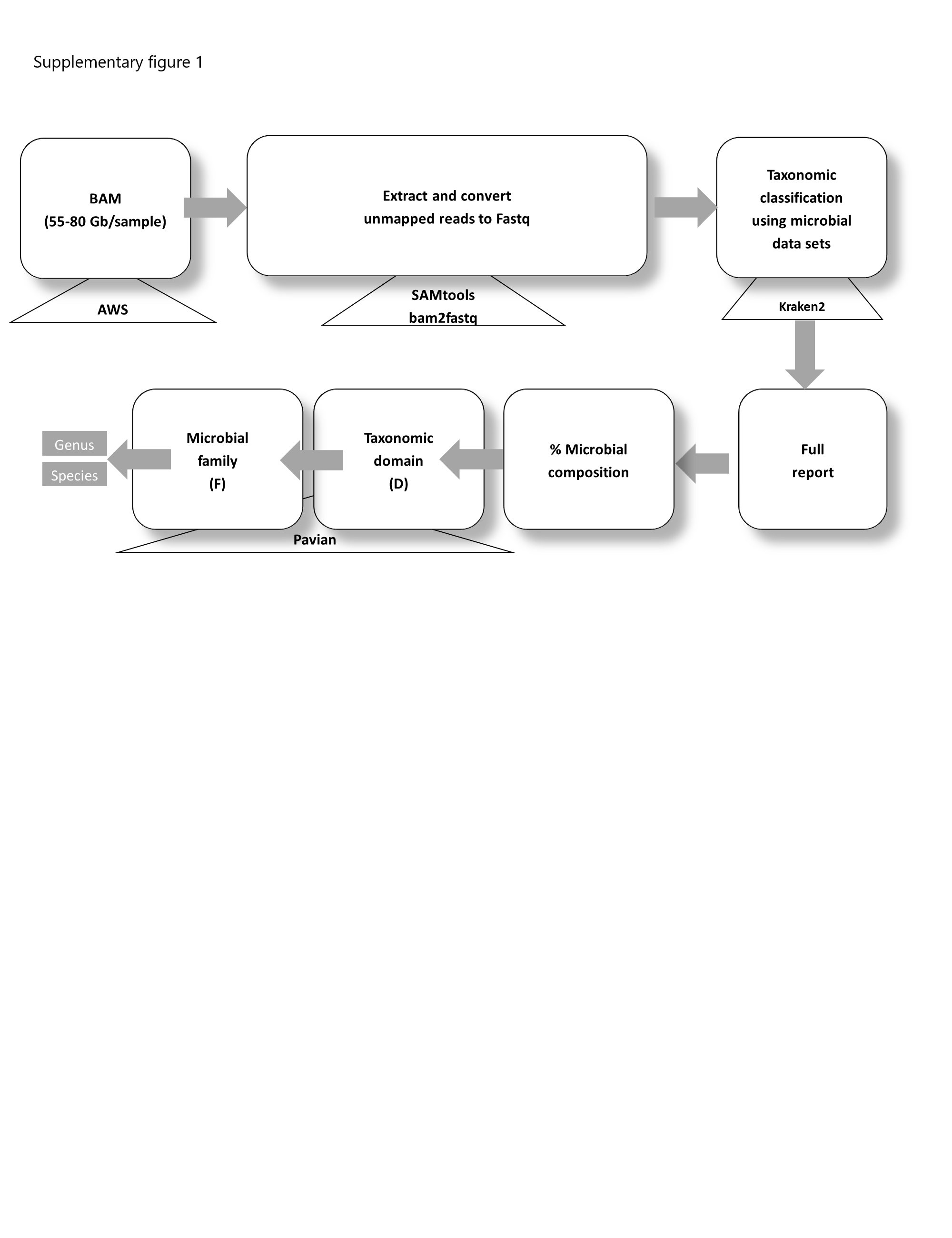

Supplement: Supplementary file 3 [file Image1.TIFF]

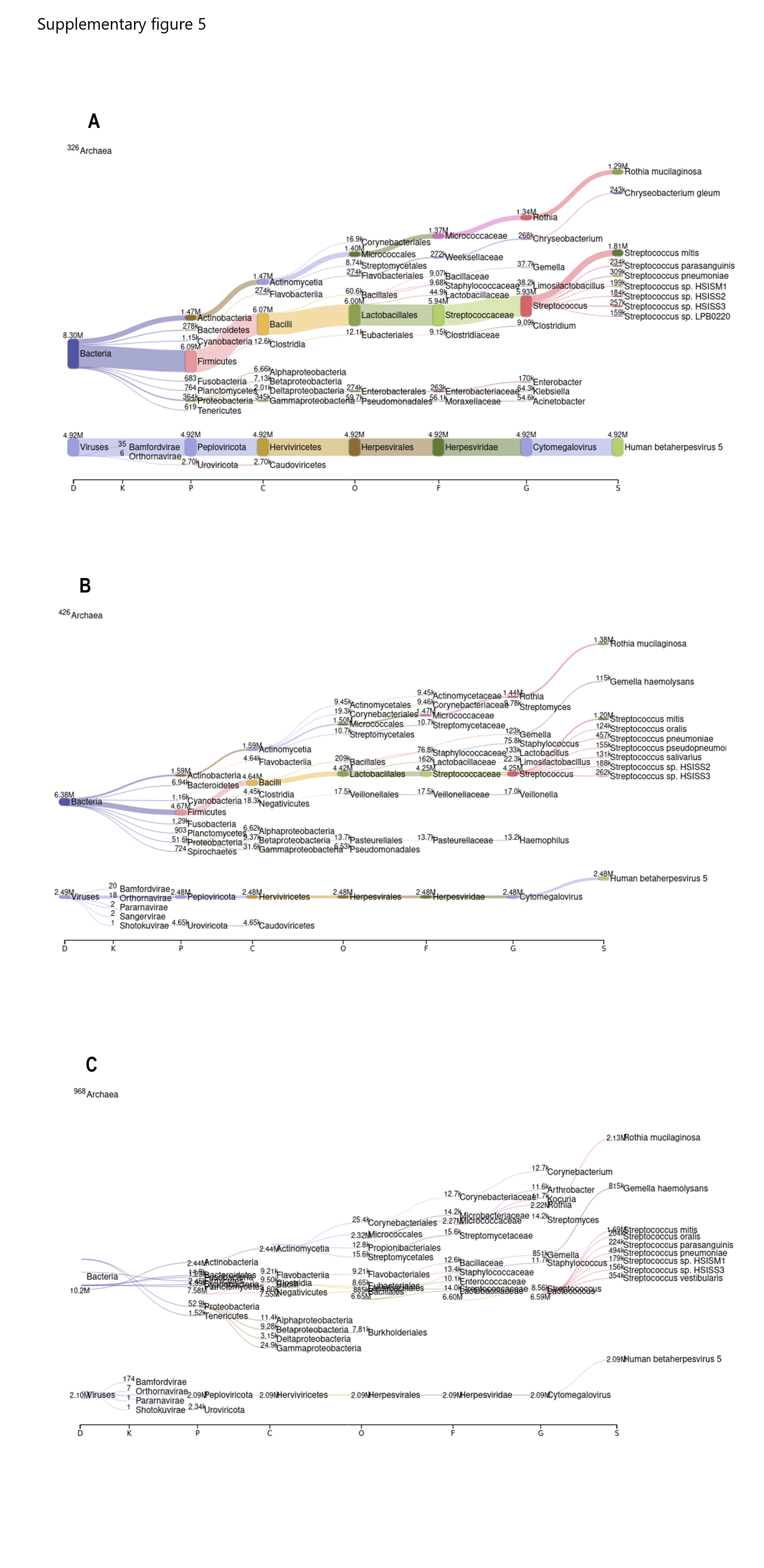

Supplement: Supplementary file 4 [file Image5.TIFF]

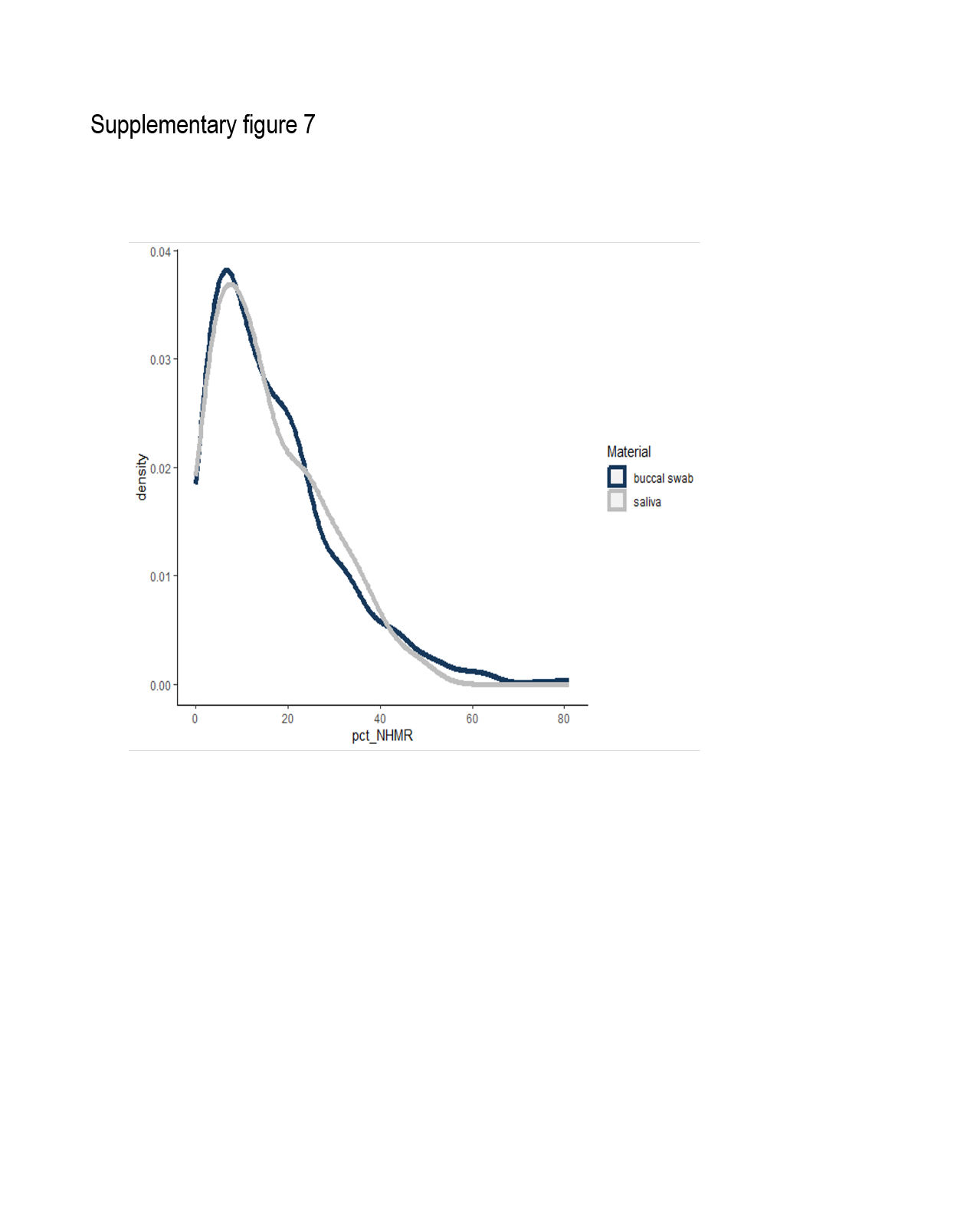

Supplement: Supplementary file 5 [file Image8.TIFF]

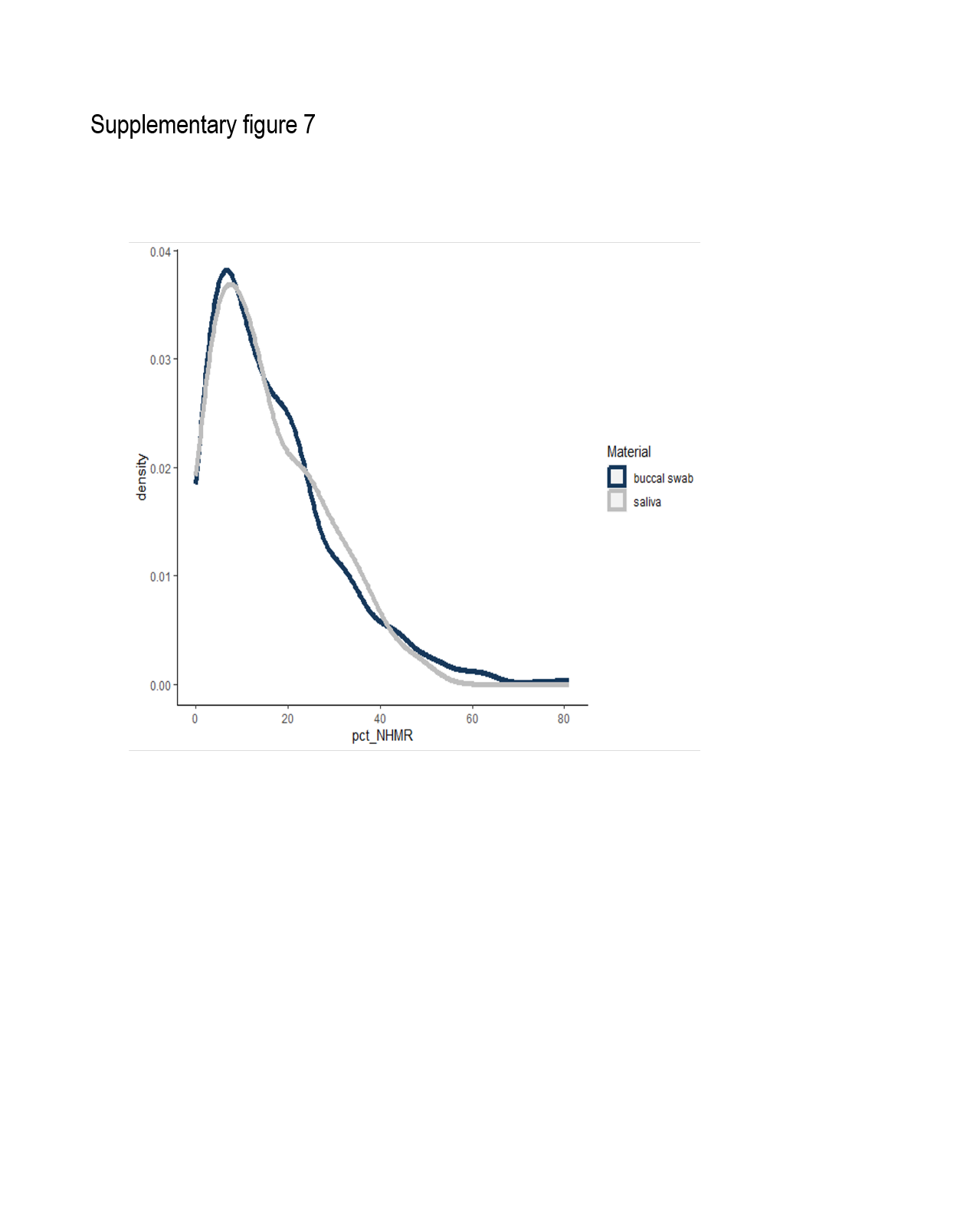

Supplement: Supplementary file 6 [file Image7.PNG]

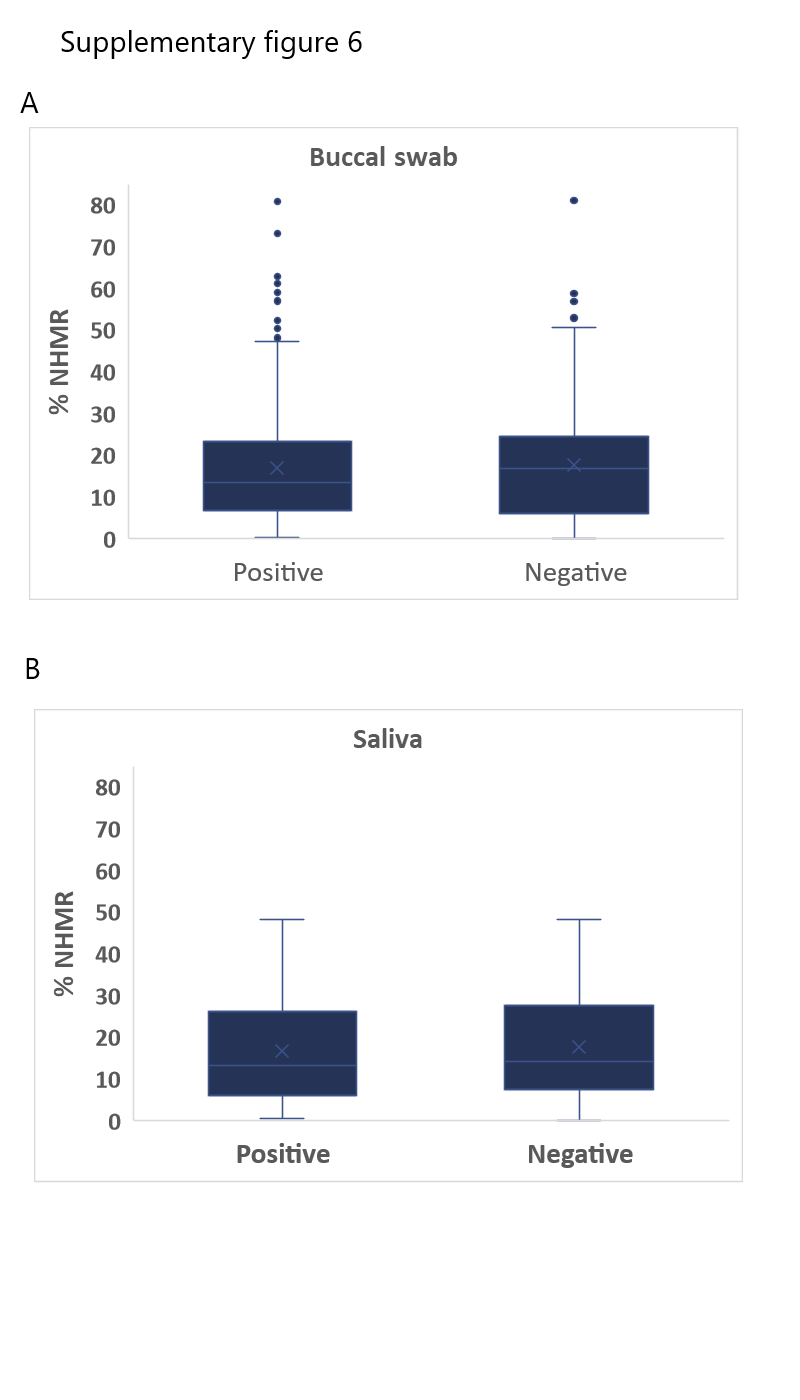

Supplement: Supplementary file 8 [file Image6.TIFF]

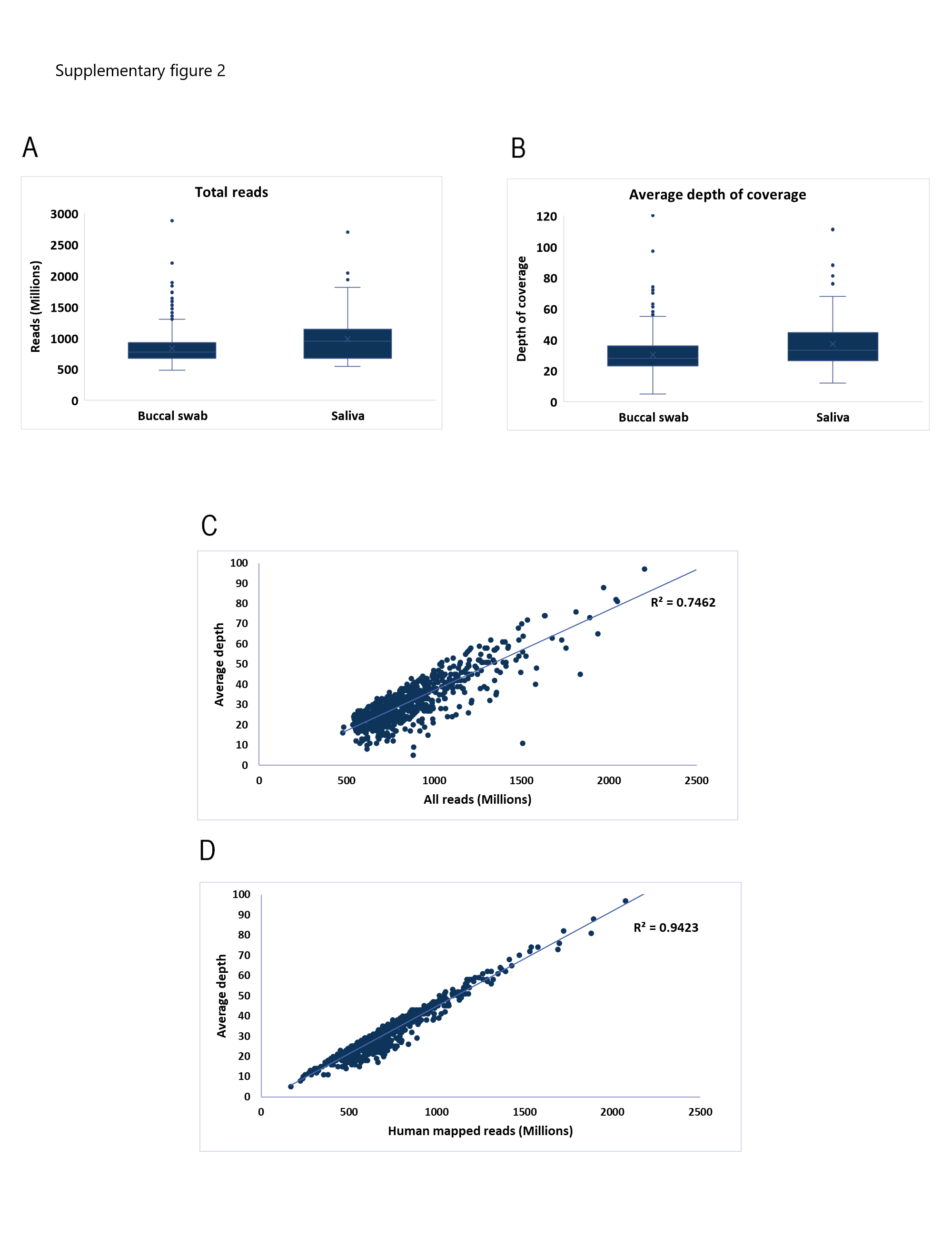

Supplement: Supplementary file 9 [file Image2.TIFF]

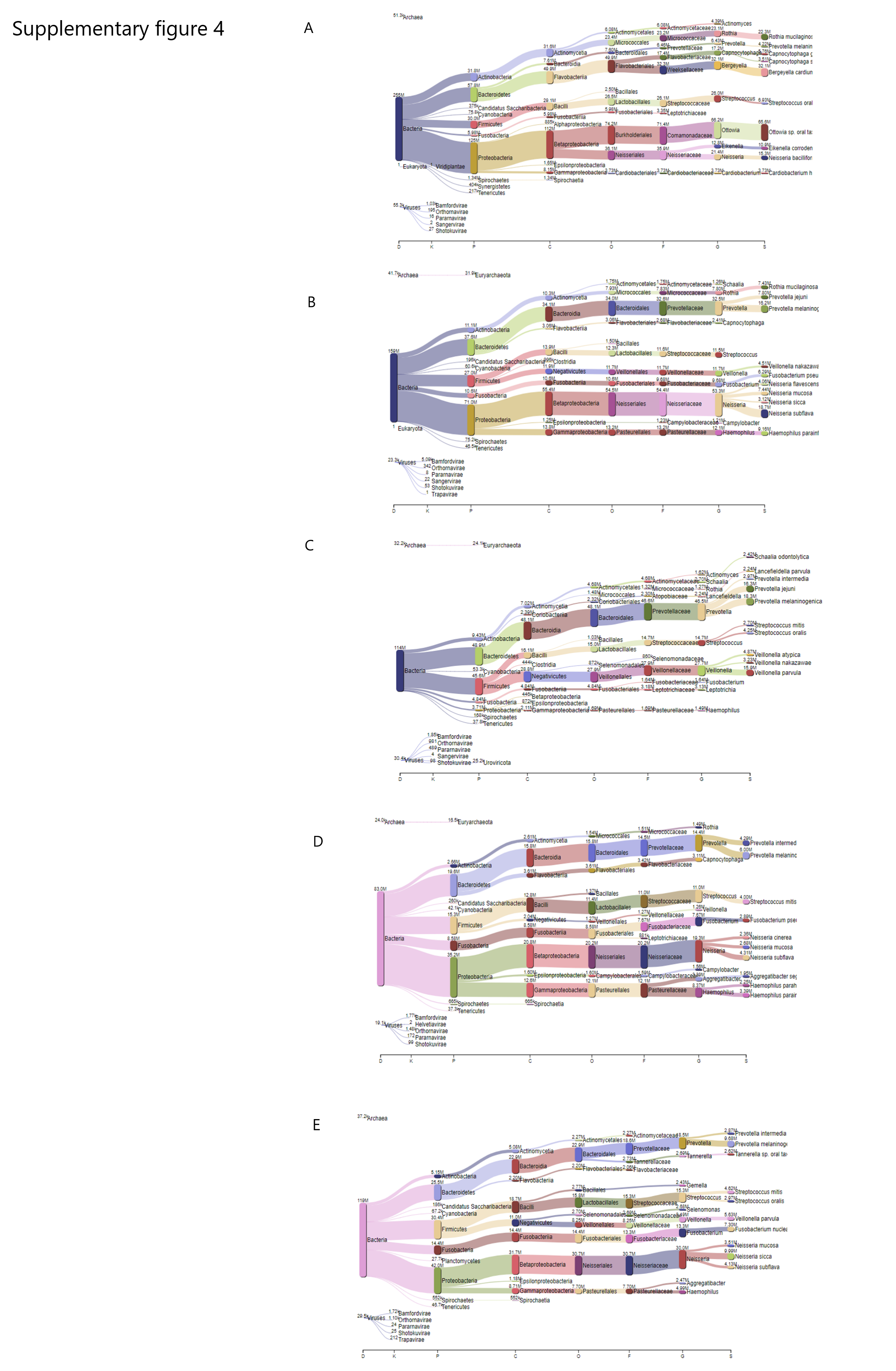

Supplement: Supplementary file 10 [file Image4.TIFF]
